# BIN1 rs744373 variant shows different association with Alzheimer’s disease in Caucasian and Asian populations

**DOI:** 10.1186/s12859-019-3264-9

**Published:** 2019-12-24

**Authors:** Zhifa Han, Tao Wang, Rui Tian, Wenyang Zhou, Pingping Wang, Peng Ren, Jian Zong, Yang Hu, Shuilin Jin, Qinghua Jiang

**Affiliations:** 1 School of Life Science and Technology, Harbin Institute of Technology, Harbin, China; 2 Department of Mathematics, Harbin Institute of Technology, Harbin, China

**Keywords:** Alzheimer’s disease, Bridging integrator 1 (BIN1), Rs744373 polymorphism, East Asian, Caucasian

## Abstract

**Background:**

The association between BIN1 rs744373 variant and Alzheimer’s disease (AD) had been identified by genome-wide association studies (GWASs) as well as candidate gene studies in Caucasian populations. But in East Asian populations, both positive and negative results had been identified by association studies. Considering the smaller sample sizes of the studies in East Asian, we believe that the results did not have enough statistical power.

**Results:**

We conducted a meta-analysis with 71,168 samples (22,395 AD cases and 48,773 controls, from 37 studies of 19 articles). Based on the additive model, we observed significant genetic heterogeneities in pooled populations as well as Caucasians and East Asians. We identified a significant association between rs744373 polymorphism with AD in pooled populations (*P* = 5 × 10^− 07^, odds ratio (OR) = 1.12, and 95% confidence interval (CI) 1.07–1.17) and in Caucasian populations (*P* = 3.38 × 10^− 08^, OR = 1.16, 95% CI 1.10–1.22). But in the East Asian populations, the association was not identified (*P* = 0.393, OR = 1.057, and 95% CI 0.95–1.15). Besides, the regression analysis suggested no significant publication bias. The results for sensitivity analysis as well as meta-analysis under the dominant model and recessive model remained consistent, which demonstrated the reliability of our finding.

**Conclusions:**

The large-scale meta-analysis highlighted the significant association between rs744373 polymorphism and AD risk in Caucasian populations but not in the East Asian populations.

## Introduction

Alzheimer’s disease (AD) is the preeminent cause for dementia in the elderly and causes 50–75% dementia types [1–3]. In the populations of Caucasian ancestry, large-scale genome-wide association studies (GWASs) have identified some common genetic risk factors for AD [4]. In addition to the APOE gene (encoding apolipoprotein E) as a major AD risk factor, recent GWASs of AD in Caucasian populations also identified several new genetic loci, including: bridging integrator 1 (BIN1), complement receptor 1 (CR1), clusterin (CLU), phosphatidylinositol binding clathrin assembly protein (PICALM), membrane-spanning 4-domains, subfamily A, member 4 (MS4A4)/membrane-spanning 4-domains, subfamily A, member 6E (MS4A6E), CD2-associated protein (CD2AP), CD33 molecule (CD33), EPH receptor A1 (EPHA1) and ATP-binding cassette transporter A7 (ABCA7) [4–7]. These genetic loci largely stimulate downstream analysis, for example the identification of relevant gene expression regulators [8–15], the construction gene expression regulatory networks [16–20], and so on.

Rs744373 is a single nucleotide polymorphism (SNP) that locates upstream of BIN1 gene. In populations of Caucasian ancestry, rs744373 polymorphism was consistently confirmed to be significantly associated with AD risk with *P* = 3.16 × 10 ^− 10^ [21], *P* = 2.6 × 10 ^− 14^ [6], *P* = 2.13 × 10 ^− 09^ [22], *P* = 2.9 × 10 ^− 07^ [23] and *P* = 1.1 × 10 ^− 04^ [24]. Recently, the association has also been extensively investigated in East Asian populations. However, besides the positive associations, many studies have also identified negative results. Tan et al. did not report any significant association when analyzd 1224 Chinese individuals (612 cases and 612 controls) using allele test (*P* = 0.217) and genotype test (*P* = 0.547, 0.263 and 0.397 for dominant, recessive and additive logistic genetic models) [25]. The result from Li et al. was also negative [26]. Wang et al. identified a significant result in population from East China (*P* = 0.038), but not southwest China (*P* = 0.874). When combining the two parts of populations, they still did not identify any significant association (*P* = 0.187) [27]. In Brazilian Chinese population, Ramos et al. analyzed 241 individuals (82 cases and 159 controls) and didn’t find any significant results (*P* = 0.660 for dominant model and *P* = 0.547 for recessive model) [28]. Ohara et al. did not report significant association (*P* = 0.06 for additive model) when analyzed 825 AD cases and 2934 controls from Japan [29]. In 2013, we conducted a meta-analysis using all currently available samples (2022 AD cases and 4209 controls) and the results were significant (*P* = 1.19 × 10 ^− 02^, 7.08 × 10 ^− 03^ and 5.75 × 10 ^− 03^ for the dominant model, recessive model and additive model) [30]. Another subsequent meta-analysis with more samples (11,832 AD cases and 18,133 controls) obtained a consistent result with us [3].

Given the inconsistent findings in East Asian populations, we believe that the relatively small sample sizes, as well as the genetic heterogeneity of AD susceptibility loci among different populations, may be important factors in the untrustworthiness of the results. In this study, we aimed to collect more studies and samples than before and obtain more statistically significant results by performing genetic heterogeneity test and meta-analysis of the rs744373 polymorphism in the Caucasians, East Asians, and pooled populations.

## Materials and methods

### Literature acquisition

In order to find all available association studies, we searched the PubMed database (https://www.ncbi.nlm.nih.gov/pubmed) and AlzGene database (http://www.alzgene.org/) with the Keywords “Alzheimer’s disease”, “Bridging Integrator 1” or “BIN1”. We also searched Google Scholar (http://scholar.google.com/) to acquire the articles citing the studies obtained in the PubMed and AlzGene databases. The literature acquisition was updated on December 12, 2017. In addition, we collected as much data as we could by directly contacting with authors. These datasets were not published due to not significant results, etc., and were not included in the previous meta-analysis of rs744373 polymorphism with AD.

### Inclusion criteria

The studies inclusion criteria contained: (1) being a case-control study; (2) investigating the association between rs744373 polymorphism and AD; (3) being conducted in East Asian or Caucasian populations; (4) providing the numbers of rs744373 genotypes or sufficient data to calculate them or (5) providing an OR with 95% confidence interval (CI) and the *P*-value or sufficient data to calculate them.

### Data extraction

The information was extracted from each study contained: (1) author names; (2) publication year; (3) the sample’s ethnicity; (4) the numbers of cases and controls; (5) the genotyping platform; (6) the frequencies of rs744373 genotypes or sufficient data to calculate them or (7) the OR with 95% CI or sufficient data to calculate them.

### Genetic model

Since not all studies provided exact genotype numbers, we investigated the association between rs744373 polymorphism and AD risk in this meta-analysis primarily using the additive genetic model. We selected allele C as effect allele and T as reference allele, the additive model can be described as C allele versus T allele [31].

### Comparison of MAF and OR in Caucasians and east Asians

We compared the minor allele frequency (MAF), which is the frequency of rs744373 allele C in this study, and the OR values between the Caucasian populations and East Asian populations. We used the t-test to investigate whether there were differences in the OR values and MAF values between these two populations. We used program R (http://www.r-project.org/) to perform the t-test and calculate the OR and MAF values that not available in the original articles.

### Heterogeneity test

We used the Cochran’s Q test to investigate genetic heterogeneity among different studies. Cochran’s Q test approximately follows a chi-square distribution and its degree of freedom is *k-1* (*k* represents the number of studies included in this studies). Statistics *I*^*2*^ can also use to measure the genetic heterogeneity, which is calculated as:

$$ {\mathrm{I}}^2=\frac{\mathrm{Q}-\left(\mathrm{k}-1\right)}{\mathrm{Q}}\times 100\% $$

The statistics *I*^*2*^ is in the range of 0–100%, and we divided it into four parts: 0–25%, 26–50%, 51–75%, 76–100%, which respectively represent low, moderate, large and extreme heterogeneity [30]. We conducted Cochran’s Q test in East Asians, Caucasians, and pooled Populations respectively. All calculations of *P*-value and *I*^*2*^ value were completed using the program R (http://www.r-project.org/). We choose *P* < 0.05 or *I*^*2*^ > 50% as discriminant criterion for significant result of heterogeneity test.

### Meta-analysis

In the meta-analysis, we used fixed effect model (Mantel–Haenszel) or random effect model (DerSimonian–Laird) to calculate the overall OR. And which model to choose depends on whether the genetic heterogeneity is significant or not. If the *P*-value of Cochran’s Q test was less than 0.05, and *I*^*2*^ value was greater than 50%, we selected the random effect model, otherwise we selected the fixed effect model. The signification of overall OR was measured by Z test.

### Sensitivity analysis and publication Bias analysis

To further test the stability of our results, we conducted a sensitivity analysis by sequentially removing each study in the meta-analysis at a time. We used funnel plots to evaluate the potential publication bias. A symmetrical inverted funnel indicated the results were no bias, and an asymmetrical inverted funnel indicated bias results [4]. Begg’s test and Egger’s test was used to evaluate the asymmetry of the funnel plot [4]. The significant level was 0.01. All statistical tests above were also performed using the program R (http://www.r-project.org/).

## Results

### Literature search and data description

**Fig. 1:**
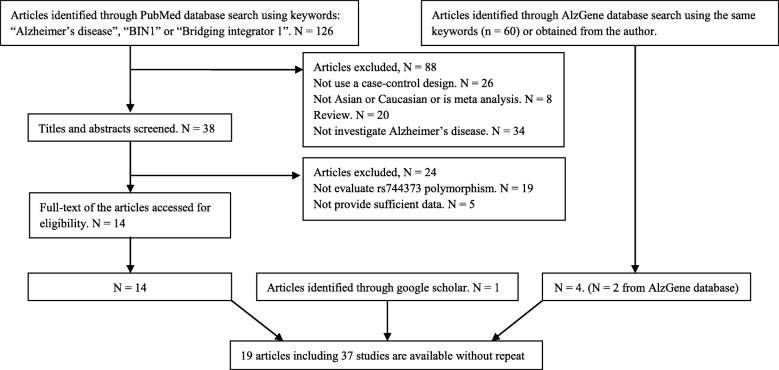
A figure Flow diagram of article inclusion or exclusion

We obtained 126 articles by searching the PubMed database. Eighty-eight articles were excluded because they were (1) not Case-Control design, (2) not analyzed in East Asian or Caucasian populations, (3) not related with AD, (4) meta-analysis or (5) review articles. We further excluded 24 articles because they did not investigate the association between rs744373 polymorphism and AD or not provide sufficient data. The remaining 14 articles met the analysis requirements. According to the same criteria, we also obtained two articles from the AlzGene database. In addition, we had found one article by searching Google Scholar. We applied for two datasets of two articles (studies) by contacting the author directly. Finally, 37 studies in 19 articles, including 22,395 AD cases and 48,773 control samples, were included in this meta-analysis. More detailed information about selecting studies was described in Fig. 1. The main characteristics of included studies were described in Table 1.

**Table 1:** Main characteristic of included studies

Study	Population/City	Ethnicity	Case			Control			MAF (C)	OR	Genotype Platform
			N	Female (%)	Age^+^ (mean ± SD)	N	Female (%)	Age^+^ (mean ± SD)			
Miyashita 2013 [32]	Japanese	East Asian	1008	72	73.0 ± 4.3	1016	57%	77.0 ± 5.9	0.33	1.11	Affymetrix GeneChip
Miyashita 2013 [32]	Koreans	East Asian	339	72	73.7 ± 9.5	1129	49%	71.0 ± 4.9	0.36	0.97	TaqMan
Xiao 2015 [33]	Chinese	East Asian	459	50.3	71.2 ± 9.6	751	52.9	72.7 ± 5.9	0.37	1.07	iPLEX
Huang 2016 [34]	Tibetans	East Asian	39	NA	NA	56	NA	NA	0.32	1.04	PCR
Jiao 2015 [35]	Chinese	East Asian	229	56.9	75.2 ± 5.0	318	53.2	71.6 ± 2.5	0.36	0.70	PCR-RFLP
Liao 2014 [35]	Chinese	East Asian	535	46.3	79.6 ± 7.9	1801	NA	NA	0.37	1.04	TaqMan
Tan 2013 [25]	Chinese	East Asian	612	52.5	80.9 ± 7.5	612	46.9	74.9 ± 6.3	0.34	0.66	TaqMan
Ohara 2012 [29]	Japanese	East Asian	825	77.1	83.2 ± 6.5	2933	56	60.2 ± 11.5	0.32	1.44	PCR-RFLP
Li 2015 [26]	Chinese	East Asian	420	54.3	67.7 ± 9.8	441	59.2	68.5 ± 9.4	0.38	2.08	PCR-RFLP
Wang 2014 [27]	Chinese (Southwest)	East Asian	333	NA	NA	334	NA	NA	0.36	1.24	PCR-RFLP
Wang 2014 [27]	Chinese (East)	East Asian	415	NA	NA	426	NA	NA	0.35	1.08	PCR-RFLP
Carrasquillo 2011 [22]	Autopsy	Caucasian	296	67.5	87.3 ± 4.8	95	52.5	85.9 ± 4.3	0.30	0.95	TaqMan
Carrasquillo 2011 [22]	Jacksonville	Caucasian	487	62.2	80.1 ± 6.5	949	56.3	81.6 ± 7.6	0.29	1.08	TaqMan
Carrasquillo 2011 [22]	Norway	Caucasian	340	70.1	80.2 ± 7.3	550	59.7	75.4 ± 7.3	0.31	1.00	TaqMan
Carrasquillo 2011 [22]	Rochester	Caucasian	310	62	85.7 ± 4.5	1619	54.6	80.3 ± 5.2	0.27	0.79	TaqMan
Carrasquillo 2011 [22]	southampton	Caucasian	35	66.7	81.2 ± 6.5	128	48.5	76.3 ± 6.3	0.32	0.94	TaqMan
Carrasquillo 2011 [22]	Bristol	Caucasian	135	58	76.9 ± 7.3	32	55	75.8 ± 6.4	0.27	1.12	TaqMan
Carrasquillo 2011 [22]	Leeds	Caucasian	113	50.4	75.1 ± 6.4	272	49.3	76.9 ± 6.2	0.29	0.94	TaqMan
Carrasquillo 2011 [22]	Man/Notts	Caucasian	173	57.9	75.8 ± 9.4	84	38.2	73.1 ± 8.3	0.35	0.99	TaqMan
Carrasquillo 2011 [22]	NCRAD	Caucasian	690	64.7	75.2 ± 6.8	202	61.7	78.3 ± 8.9	0.30	1.00	TaqMan
Carrasquillo 2011 [22]	Oxford	Caucasian	98	49	73 ± 7.2	203	57.1	77.2 ± 8	0.31	0.89	TaqMan
Carrasquillo 2011 [22]	Poland	Caucasian	468	66.2	76.7 ± 4.8	180	77.2	73.0 ± 5.9	0.28	1.00	TaqMan
Lambert 2011 [24]	Finland	Caucasian	563	68	71.3 ± 7.4	529	58	69.0 ± 6.4	0.24	0.90	TaqMan
Lambert 2011 [24]	Italy	Caucasian	1460	68	76.6 ± 8.7	1265	55	72.3 ± 8.9	0.28	0.89	TaqMan
Lambert 2011 [24]	Spain	Caucasian	726	57	75.3 ± 9.3	829	62	76.9 ± 10.9	0.29	0.53	TaqMan
Harold 2009 [5]	Ireland	Caucasian	2227	65	NA	4697	53	NA	0.29	0.85	Illumina platforms
Harold 2009 [5]	Germany	Caucasian	555	64	NA	824	51	NA	0.30	0.89	Illumina platforms
Harold 2009 [5]	USA	Caucasian	551	58	NA	929	56	NA	0.29	1.00	Illumina platforms
Ramos 2016 [28]	Brazilian	Caucasian	82	65.9	81.2 ± 7.5	159	73.0	79.2 ± 7.8	0.34	0.98	PCR-RFLP
Gharesouran 2014 [36]	Iran	Caucasian	160	58.8	76.1 ± 7.8	163	58.28	75.3 ± 6.8	0.09	0.82	PCR-RFLP
Hu 2011 [23]	USA	Caucasian	1034	NA	NA	1186	NA	NA	0.30	0.88	Illumina 610Quad, HumanHap550
Carrasquillo 2014 [37]	USA	Caucasian	54	76	61.3 ± 9.2	2397	NA	NA	0.27	0.95	TaqMan
Seshadri 2010 [38]	white people	Caucasian	3006	NA	NA	14,642	NA	NA	0.29	0.93	Various Illumina chips, Affymetrix GeneChip
Seshadri 2010 [38]	France	Caucasian	2032	NA	NA	5328	NA	NA	0.29	0.84	Illumina Human 610Quad
Seshadri 2010 [38]	Spain	Caucasian	1140	69.9	78.8 ± 7.9	1209	52.8	49.9 ± 9.2	0.28	0.88	PCR-RFLP
Nizamutdinov 2013 [39]	Moscow	Caucasian	166	NA	NA	128	NA	NA	0.33	0.90	Biochip
Moreno 2017 [40]	Colombian	Caucasian	280	76.1	75.5 ± 7.2	357	73.9	71.0 ± 7.1	0.29	1.12	PCR-RFLP

N, Number of subjects; SD, Standard Deviation, MAF (C), Minor Allele (allele C of rs744373) Frequency; OR, odds ratio

^+^ Do not distinguish “Age at onset” or “age at exam (study)”

### Comparison of MAF and OR between Caucasian and east Asian

There were 11 studies belong to East Asian populations. The MAF values of rs744373, OR values and other information of these 11 studies listed in the top 11 rows in Table 1. The other studies listed in the last 26 rows in Table 1 belonged to Caucasian populations. By using the t-test to compare the MAF values between Caucasians and East Asians, we found a significant result with *t* = 5.89 and *P* = 1.53 × 10^− 6^. However, the result of comparison of OR values did not indicate a very significant distinction between the two populations (*t* = 1.75 and *P* = 0.11).

### Heterogeneity test

**Fig. 2:**
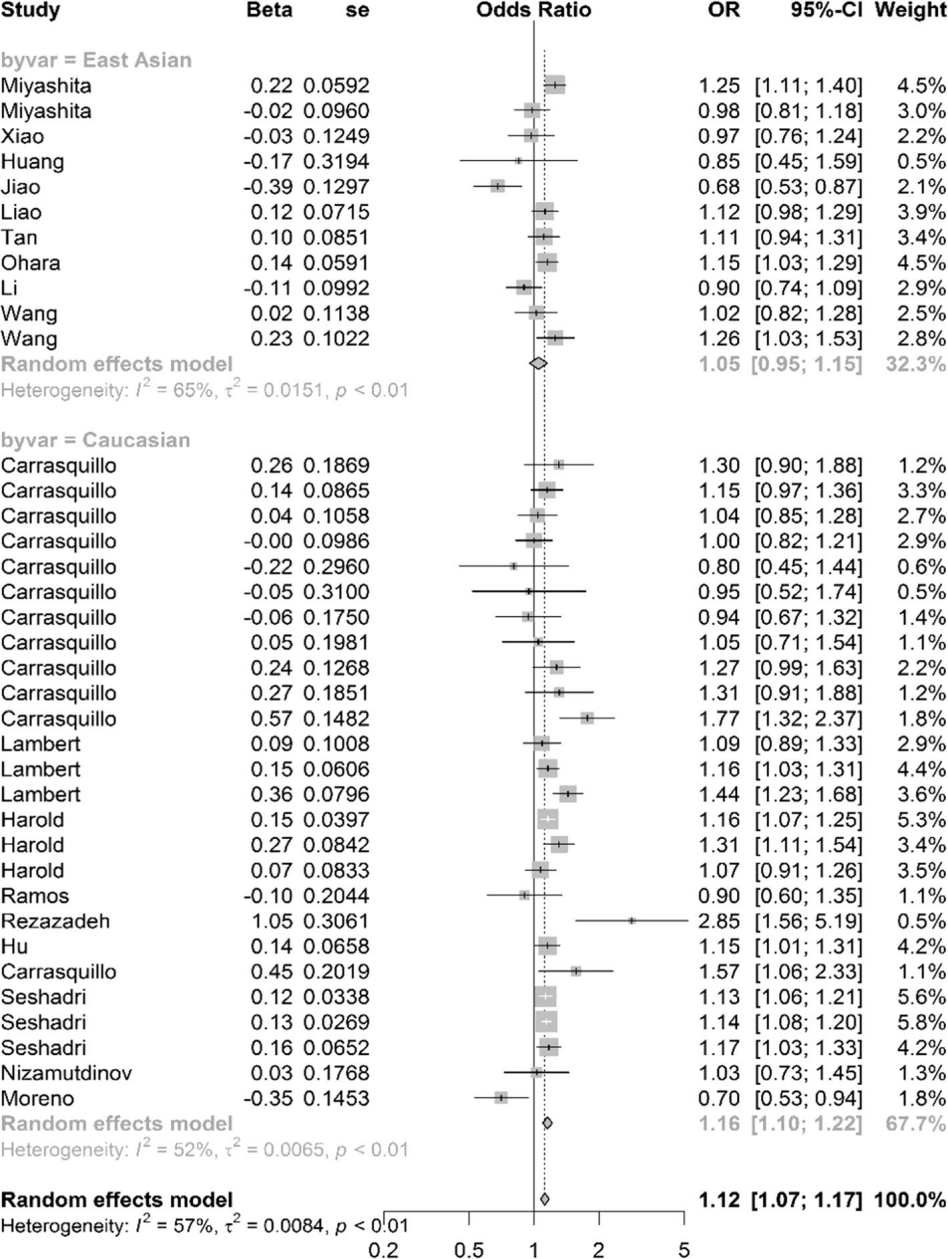
Forest plot for the meta-analysis of the association between rs744373 and AD under the additive model. “OR” is the abbreviation of Odds Ratio. “Beta” indicates the ln (OR). “se” is the standard error of Beta. “Weight” represents the weight of each study when calculating the overall OR. The genetic heterogeneity test results (*I*^*2*^ and its *P*-value) and the meta-analysis results (overall OR and 95% CI) in pooled populations are listed at the bottom of the figure. The results for subgroup analysis are also listed by the grey font

We conducted heterogeneity test of rs744373 polymorphism in different populations, and identified significant genetic heterogeneity in Caucasians (*P* = 0.001, *I*^*2*^ = 52.3%), East Asians (*P* = 0.001, *I*^*2*^ = 65.1%) and pooled populations (*P* = 1.03 × 10^− 5^, *I*^*2*^ = 57.2%). Detailed results were described in Table 2 and Fig. 2.

**Table 2:** The results of genetic heterogeneity test and meta-analysis of rs744373 polymorphism in East Asian and Caucasian populations

	East Asian	Caucasian	East Asian VS Caucasian
*I*^*2*^ for heterogeneity test	0.651	0.523	0.572
*P* for heterogeneity test	0.001	0.001	1.03E-05
OR for meta-analysis	1.05	1.16	1.12
95%CI for meta-analysis	0.95–1.15	1.10–1.22	1.07–1.17
*P* for meta-analysis	0.331	3.38E-08	5.00E-07

### Meta-analysis

Based on the results of Heterogeneity test, we used random effect model to calculate the overall OR values in East Asians, Caucasians and pooled populations, respectively. Meta-analysis results indicated significant correlation in Caucasians with *P* = 3.38 × 10^− 08^, OR = 1.16, 95% CI 1.10–1.22, and in pooled populations with *P* = 5 × 10^− 07^, OR = 1.12, and 95% CI 1.07–1.17 (Table 2). However, we did not find any association between rs744373 polymorphism and AD in East Asian populations with *P* = 0.39, OR = 1.06, and 95% CI 0.95–1.15. The detailed results and forest diagram were described in Table 2 and Fig. 2.

### Sensitivity analysis and publication Bias analysis

**Fig. 3:**
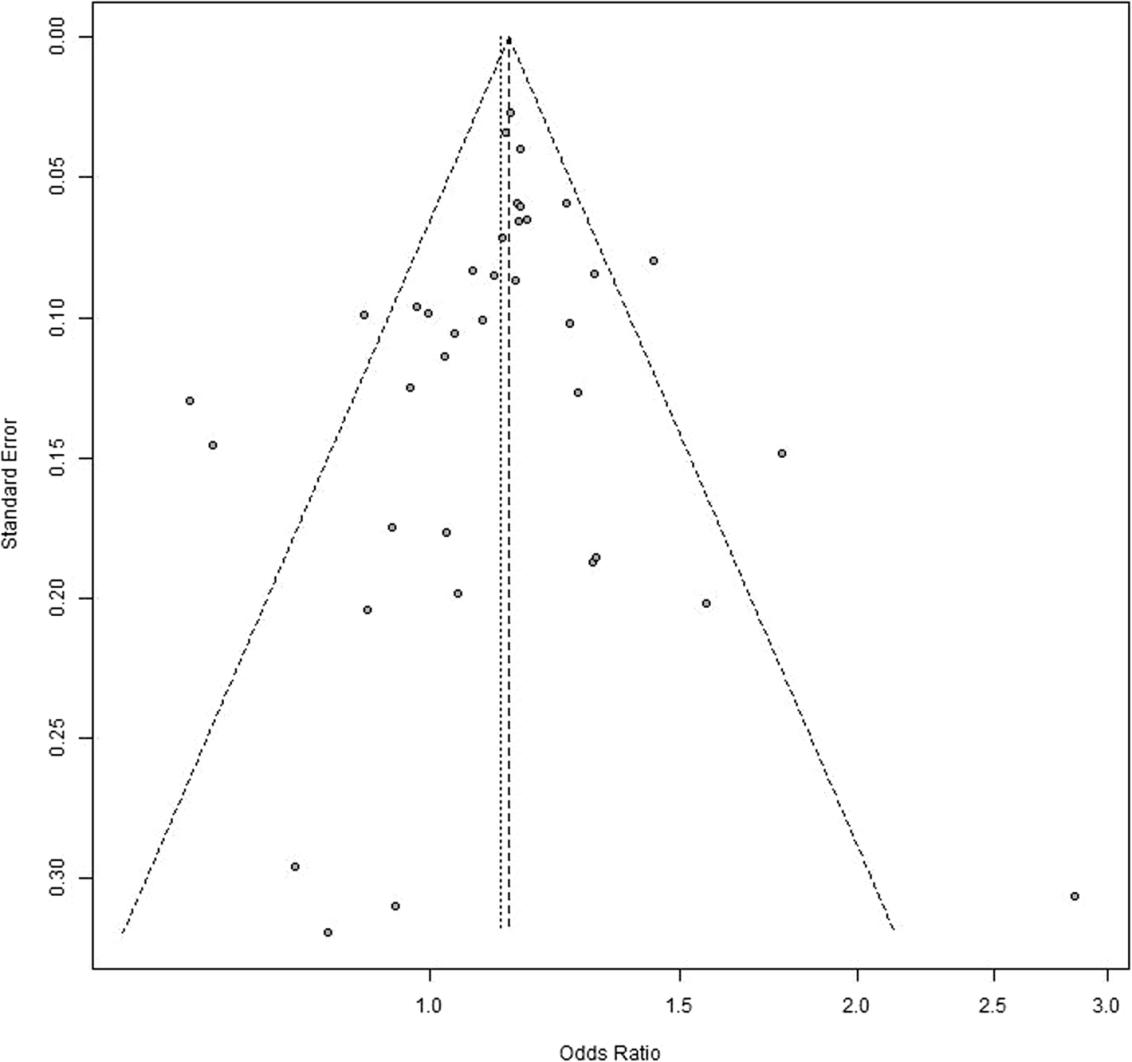
Analysis of publication bias regarding the rs744373 polymorphism in AD. The symmetrical inverted funnel shows that there is not publication bias

Using sensitivity analysis, we identified that the results of meta-analysis remained largely unchanged by excluding any one study (Table 3). The symmetrical inverted funnel in the funnel plot suggested no publication bias of the results (Begg’s test, *P* = 0.471; Egger’s test, *P* = 0.428). Funnel diagram was described in Fig. 3.

**Table 3:** The results of sensitivity analysis

Excluded study	*I*^*2*^	*P* for heterogeneity test	OR under random effect model	95%CI of OR	*P* for meta-analysis
Miyashita 2013 [32]	0.57	1.48E-05	1.117	1.066–1.17	2.98E-06
Miyashita 2013 [32]	0.57	1.38E-05	1.128	1.077–1.181	2.64E-07
Xiao 2015 [33]	0.58	1.08E-05	1.127	1.076–1.179	3.15E-07
Huang 2016 [34]	0.58	8.45E-06	1.124	1.075–1.177	4.09E-07
Jiao 2015 [35]	0.49	0.000699	1.137	1.09–1.185	1.40E-09
Liao 2014 [35]	0.58	6.55E-06	1.122	1.071–1.176	1.35E-06
Tan 2013 [25]	0.58	6.66E-06	1.123	1.072–1.177	1.05E-06
Ohara 2012 [29]	0.58	6.60E-06	1.121	1.069–1.175	2.07E-06
Li 2015 [26]	0.55	3.74E-05	1.131	1.081–1.183	7.33E-08
Wang 2014 [27]	0.58	8.46E-06	1.125	1.075–1.178	4.79E-07
Wang 2014 [27]	0.58	8.75E-06	1.119	1.069–1.172	1.71E-06
Carrasquillo 2011 [22]	0.58	7.67E-06	1.121	1.071–1.173	1.01E-06
Carrasquillo 2011 [22]	0.58	6.54E-06	1.122	1.071–1.175	1.35E-06
Carrasquillo 2011 [22]	0.58	8.11E-06	1.125	1.074–1.178	5.43E-07
Carrasquillo 2011 [22]	0.58	1.12E-05	1.127	1.076–1.18	3.46E-07
Carrasquillo 2011 [22]	0.58	9.92E-06	1.125	1.075–1.177	3.37E-07
Carrasquillo 2011 [22]	0.58	7.24E-06	1.124	1.074–1.176	5.01E-07
Carrasquillo 2011 [22]	0.58	9.40E-06	1.126	1.076–1.178	3.50E-07
Carrasquillo 2011 [22]	0.58	6.86E-06	1.124	1.073–1.176	5.85E-07
Carrasquillo 2011 [22]	0.58	8.34E-06	1.12	1.069–1.172	1.40E-06
Carrasquillo 2011 [22]	0.58	7.80E-06	1.121	1.071–1.173	1.02E-06
Carrasquillo 2011 [22]	0.53	9.61E-05	1.115	1.068–1.165	8.59E-07
Lambert 2011 [24]	0.58	6.88E-06	1.124	1.073–1.177	8.08E-07
Lambert 2011 [24]	0.58	6.73E-06	1.121	1.069–1.175	2.12E-06
Lambert 2011 [24]	0.53	9.42E-05	1.114	1.065–1.164	1.85E-06
Harold 2009 [5]	0.58	7.09E-06	1.12	1.067–1.175	3.96E-06
Harold 2009 [5]	0.57	1.54E-05	1.117	1.067–1.169	2.28E-06
Harold 2009 [5]	0.58	7.63E-06	1.124	1.073–1.178	7.49E-07
Ramos 2016 [28]	0.58	9.65E-06	1.126	1.076–1.178	3.35E-07
Gharesouran 2014 [36]	0.53	9.70E-05	1.119	1.072–1.168	3.23E-07
Hu 2011 [23]	0.58	6.61E-06	1.121	1.07–1.175	1.85E-06
Carrasquillo 2014 [37]	0.57	1.42E-05	1.119	1.07–1.171	1.02E-06
Seshadri 2010 [38]	0.58	6.58E-06	1.121	1.068–1.177	4.30E-06
Seshadri 2010 [38]	0.58	6.52E-06	1.12	1.066–1.177	8.02E-06
Seshadri 2010 [38]	0.58	6.91E-06	1.12	1.069–1.174	2.07E-06
Nizamutdinov 2013 [39]	0.58	7.20E-06	1.124	1.074–1.177	5.41E-07
Moreno 2017 [40]	0.52	0.000171	1.133	1.086–1.183	1.06E-08

## Discussion

GWASs showed that SNPs located in upstream of BIN1, particular rs744373, are strongly associated with AD risk [41]. The expression quantitative trait loci (eQTL) analysis identified a pronounced association between rs744373 and the expression of BIN1 in brain tissue [3]. BIN1 gene have diverse functions, including endocytosis, trafficking, immune response, apoptosis, and tau metabolism, that are thought have potential roles in AD pathological mechanism [41, 42]. To some extent, investigating the association between rs744373 and AD risk is helpful for understanding the role of BIN1 in AD pathogenesis.

Based on the significant association between rs744373 polymorphism and AD risk identified by the GWASs in Caucasian populations, many recent studies had also explored this association in East Asian populations, as described in the introduction. However, the findings of the association studies in East Asian were always inconsistent. Considering a relatively small sample size may result in less statistical power, we collected 37 studies involving 22,395 AD cases and 48,773 controls for the meta-analysis. To the best of our knowledge, this was the largest sample size by far.

By meta-analysis of the 37 studies, we obtained significant association between rs744373 polymorphism and AD risk in pooled populations (*P* = 5 × 10^− 07^, OR = 1.12, and 95% CI 1.07–1.17) and also in Caucasian populations (*P* = 3.38 × 10^− 08^, OR = 1.16, 95% CI 1.10–1.22). The results were consistent with the previous studies. However, in East Asian populations, our results showed a significant genetic heterogeneity of rs744373 polymorphism (*P* = 0.001, *I*^*2*^ = 65.1%) and the meta-analysis did not show a significant association between rs744373 polymorphism with AD risk by using a random effect model (*P* = 0.393, OR = 1.057, and 95% CI 0.95–1.15). The insignificant publication bias results and the consistent sensitivity analysis results showed that our results were reliable.

**Fig. 4:**
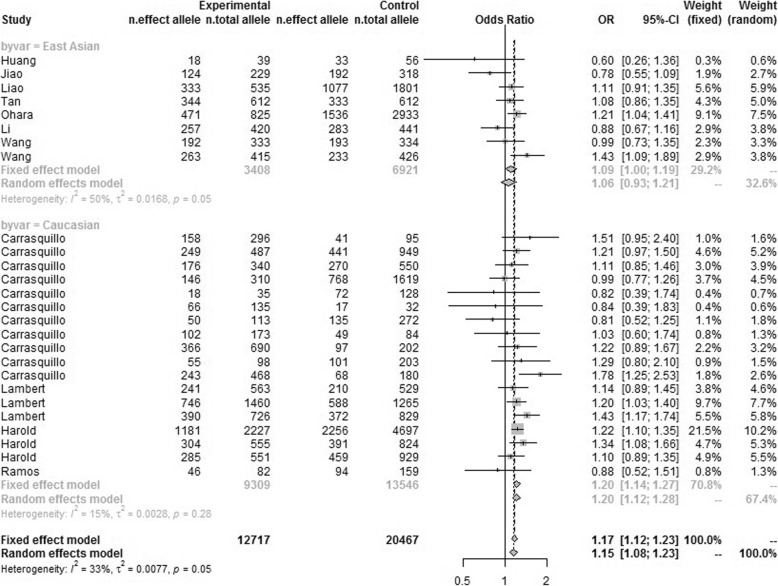
Forest plot for the meta-analysis of the association between rs744373 and AD under the dominant model

**Fig. 5:**
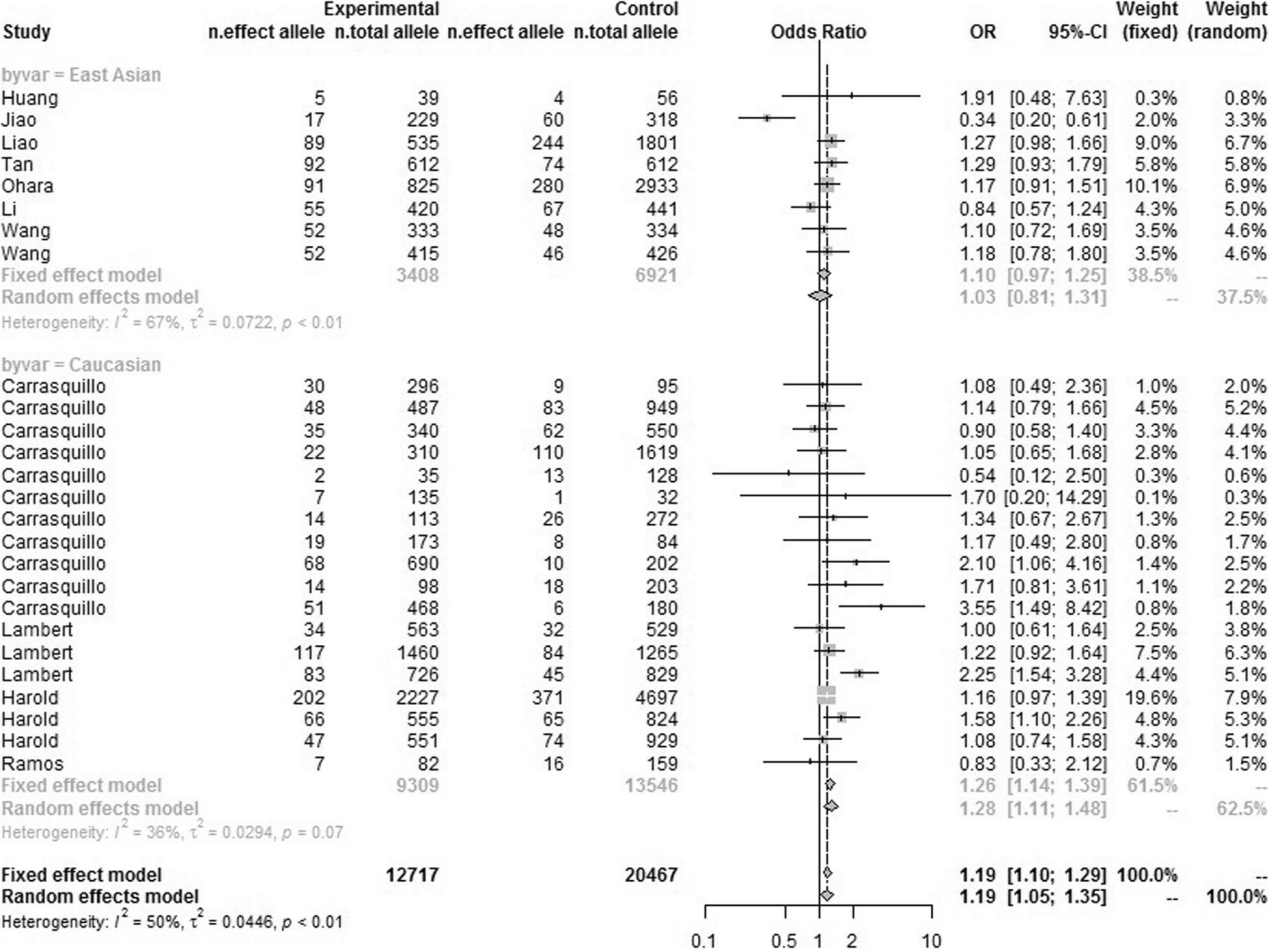
Forest plot for the meta-analysis of the association between rs744373 and AD under the recessive model

To confirm the findings that were obtained by additive genetic model, we further used the dominant model (CC + CT versus TT) and recessive model (CC versus CT + TT) to investigate the association of rs744373 polymorphism with AD risk based on genotype data of 33,184 samples (12,717 AD cases and 20,467 controls). As same as the results of additive model, we obtained significant association between rs744373 and AD in pooled populations (*P* = 3.95 × 10^− 11^, OR = 1.17, 95% CI 1.12–1.23 for dominant model and *P* = 1.35 × 10^− 05^, OR = 1.19, 95% CI 1.10–1.29 for recessive model), as well as in Caucasian populations (*P* = 5.99 × 10^− 11^, OR = 1.20, 95% CI 1.14–1.27 for dominant model and *P* = 1.00 × 10^− 05^, OR = 1.26, 95% CI 1.14–1.39 for recessive model). We also obtained negative results in East Asian populations (*P* = 0.391, OR = 1.06, 95% CI 0.93–1.21 for dominant model and *P* = 0.806, OR = 1.03, 95% CI 0.81–1.31 for recessive model). The consistent results among the three kinds of genetic models demonstrated the reliability of our results. The data was described in Additional file 1 and the detailed results were described in Table 2, Table 4, Fig. 4, Fig. 5 and Additional file 1. The information about the samples and publication bias was described in Additional file. In summary, this large-scale meta-analysis highlighted the significant association between rs744373 polymorphism and AD in Caucasian populations but not in the East Asian populations.

**Table 4:** The genetic heterogeneity test and meta-analysis of rs744373 polymorphism using the additive model, dominant model and recessive model in East Asian and Caucasian populations

Comparisons	East Asian				Caucasian				East Asian VS Caucasian			
	*I*^*2*^	OR	95% CI	*P* for meta	*I*^*2*^	OR	95% CI	*P* for meta	I^2^	OR	95% CI	*P* for meta
C VS T	0.665	1.03	0.92–1.16	0.611	0.402	1.17	1.12–1.22	1.35E-12	0.541	1.12	1.06–1.19	0.000179
CC + CT VS TT	0.5	1.06	0.93–1.21	0.391	0.149	1.2	1.14–1.27	5.99E-11	0.33	1.17	1.12–1.23	3.95E-11
CC VS CT + TT	0.665	1.03	0.81–1.31	0.806	0.356	1.26	1.14–1.39	0.00001	0.499	1.19	1.10–1.29	1.35E-05

Researchers have begun to focus on AD genetic heterogeneity between different races and ethnicities since the end of the last century [43]. They found that the frequency variations in ApoE subtypes existed among nine populations include Caucasians and East Asians [43]. Besides the most consistent genetic risk factor ApoE for Sporadic AD, some studies have also reported many genetic risk factors that appear distinct AD susceptibility between Caucasian and East Asian populations. For instance, following genes were proven to be only associated with AD risk in Caucasian populations but not in East Asian populations: Triggering Receptor Expressed On Myeloid Cells 2 (TREM2) [44, 45], Solute Carrier Family 24 Member 4 (SLC24A4) [46], NME/NM23 Family Member 8 (NME8) [47], GRB2 Associated Binding Protein 2 (GAB2) [48], Myocyte Enhancer Factor 2C (MEF2C) [49], Inositol Polyphosphate-5-Phosphatase D (INPP5D) [50], CLU [51], ABCA7, CD2AP, and EPHA1 [25], Fermitin Family Member 2 (FERMT2) [52]. Hence, the complex difference among different ethnicities and races probably cause the genetic heterogeneity of AD between Caucasians and East Asians.

Our samples of East Asian ancestry mainly came from Chinese, Japanese and Koreans populations. On the one hand, these samples may not be able to represent the East Asian populations completely. On the other hand, the specific differences in sample collection processes of different studies would lead to genetic heterogeneity among different populations. Considering these limitations, we believe that a large sample size GWAS in East Asian population is very necessary.

## Conclusions

Until now, the genetic association between BIN1 rs744373 and AD risk in East Asian populations is still not deterministic. In the study, we conducted a meta-analysis with the largest sample size so far (22,395 AD cases and 48,773 controls). The meta-analysis results under the additive, dominant and recessive model indicated a significant association between rs744373 and AD risk in Caucasian populations but not in East Asian populations. The consistent results of sensitivity analysis, as well as the negative results of publication bias analysis, demonstrated the reliability of our findings. We believe that the greater statistically powerful results are helpful for the understanding of AD genetic risk factors in East Asian populations.

## Supplementary information

**Additional file 1. **Meta-analysis under dominant and recessive model. **Table S1.** The selected studies investigating the association between rs744373 and AD using dominant model and recessive model **Figure S1.** Funnel plot of the publication bias analysis under dominant model. **Figure S2.** Funnel plot of the publication bias analysis under recessive model.

## Data Availability

Most of the summary statistics extracted from each study are included within the articles and its Additional files.

## Abbreviations

ABCA7: ATP-binding cassette transporter A7
AD: Alzheimer’s disease
APOE: apolipoprotein E
BIN1: bridging integrator 1
CD2AP: CD2-associated protein
CD33: CD33 molecule
CI: confidence interval
CLU: clusterin
CR1: complement receptor 1
EPHA1: EPH receptor A1
GTEx: Genotype-tissue Expression
GWAS: Genome-wide association study
MAF: minor allele frequency
MS4A4: membrane-spanning 4-domains, subfamily A, member 4
MS4A6E: membrane-spanning 4-domains, subfamily A, member 6E
OR: odds ratio
PICALM: phosphatidylinositol binding clathrin assembly protein
SE: standard error
SNP: single nucleotide polymorphism
