# Supplementary material for: BIN1 rs744373 variant shows different association with Alzheimer’s disease in Caucasian and Asian populations
Source: BMC Bioinformatics. 2019 Dec 24;20(Suppl 25):691. doi: 10.1186/s12859-019-3264-9 (PMC6929404; doi:10.1186/s12859-019-3264-9)
Supplement: Supplementary file 1 — Additional file 1. Meta-analysis under dominant and recessive model. Table S1. The selected studies investigating the association between rs744373 and AD using dominant model and recessive model Figure S1. Funnel plot of the publication bias analysis under dominant model. Figure S2. Funnel plot of the publication bias analysis under recessive model. [file 12859_2019_3264_MOESM1_ESM.docx]

**Meta-analysis under dominant and recessive model**

1. Data Description

In addition to the Additive genetic model, we further investigated the association between rs744373 polymorphism with AD risk using dominant genetic model and recessive genetic model. Just as the additive model can be described as C allele versus T allele, the dominant model can be described as CC + CT versus TT and the recessive model can be described as CC versus CT + TT. Because most of the original association studies only used the additive model, we need the genotype data to calculate the OR values for the dominant and recessive model. From the total 37 studies as descripted in Table1, we selected 26 studies that providing genotype data. The 26 studies were from 11 articles and included 33,184 samples (12,717 AD cases and 20,467 controls). The detailed genotype information about the selected studies were described in Table S1.

**Table S1.** The selected studies investigating the association between rs744373 and AD using dominant model and recessive model

| Study | Population | Case genotypes, n | | |  | Control Genotypes, n | | |
| --- | --- | --- | --- | --- | --- | --- | --- | --- |
|  |  | CC | CT | TT |  | CC | CT | TT |
| Huang 2016 [[1](#_ENREF_1)] | Tibetans | 5 | 13 | 21 |  | 4 | 29 | 23 |
| Jiao 2015 [[2](#_ENREF_2)] | Chinese | 17 | 107 | 105 |  | 60 | 132 | 126 |
| Liao 2014 [[3](#_ENREF_3)] | Chinese | 89 | 244 | 202 |  | 244 | 833 | 724 |
| Tan 2013 [[4](#_ENREF_4)] | Chinese | 92 | 252 | 268 |  | 74 | 259 | 279 |
| Ohara 2012 [[5](#_ENREF_5)] | Japanese | 91 | 380 | 354 |  | 280 | 1256 | 1397 |
| Li 2015 [[6](#_ENREF_6)] | Chinese | 55 | 202 | 163 |  | 67 | 216 | 158 |
| Wang 2014 [[7](#_ENREF_7)] | Chinese(Southwest) | 52 | 140 | 141 |  | 48 | 145 | 141 |
| Wang 2014 [[7](#_ENREF_7)] | Chinese(east) | 52 | 211 | 152 |  | 46 | 187 | 193 |
| Carrasquillo 2011 [[8](#_ENREF_8)] | Autopsy | 30 | 128 | 138 |  | 9 | 32 | 54 |
| Carrasquillo 2011 [[8](#_ENREF_8)] | Jacksonville | 48 | 201 | 238 |  | 83 | 358 | 508 |
| Carrasquillo 2011 [[8](#_ENREF_8)] | Norway | 35 | 141 | 164 |  | 62 | 208 | 280 |
| Carrasquillo 2011 [[8](#_ENREF_8)] | Rochester | 22 | 124 | 164 |  | 110 | 658 | 851 |
| Carrasquillo 2011 [[8](#_ENREF_8)] | southampton | 2 | 16 | 17 |  | 13 | 59 | 56 |
| Carrasquillo 2011 [[8](#_ENREF_8)] | Bristol | 7 | 59 | 69 |  | 1 | 16 | 15 |
| Carrasquillo 2011 [[8](#_ENREF_8)] | Leeds | 14 | 36 | 63 |  | 26 | 109 | 137 |
| Carrasquillo 2011 [[8](#_ENREF_8)] | Man/Notts | 19 | 83 | 71 |  | 8 | 41 | 35 |
| Carrasquillo 2011 [[8](#_ENREF_8)] | NCRAD | 68 | 298 | 324 |  | 10 | 87 | 105 |
| Carrasquillo 2011 [[8](#_ENREF_8)] | Oxford | 14 | 41 | 43 |  | 18 | 83 | 102 |
| Carrasquillo 2011 [[8](#_ENREF_8)] | Poland | 51 | 192 | 225 |  | 6 | 62 | 112 |
| Lambert 2011 [[9](#_ENREF_9)] | Finland | 34 | 207 | 322 |  | 32 | 178 | 319 |
| Lambert 2011 [[9](#_ENREF_9)] | Italy | 117 | 629 | 714 |  | 84 | 504 | 677 |
| Lambert 2011 [[9](#_ENREF_9)] | Spain | 83 | 307 | 336 |  | 45 | 327 | 457 |
| Harold 2009 [[10](#_ENREF_10)] | Ireland | 202 | 979 | 1046 |  | 371 | 1885 | 2441 |
| Harold 2009 [[10](#_ENREF_10)] | Germany | 66 | 238 | 251 |  | 65 | 326 | 433 |
| Harold 2009 [[10](#_ENREF_10)] | USA | 47 | 238 | 266 |  | 74 | 385 | 470 |
| Ramos 2016 [[11](#_ENREF_11)] | Brazilian | 7 | 39 | 36 |  | 16 | 78 | 65 |

1. Results for the dominant model

For the dominant model, we did not identify any significant genetic heterogeneity among pooled populations with *I^2^* = 33% and *P* = 0.054. Therefore, we calculated the OR value using fixed effect model. The results of meta-analysis indicated a significant association between rs744373 and AD risk in pooled populations with *P* = 3.95 × 10^-11^, OR = 1.17, and 95% CI 1.12-1.23 (Table 3). We did not identify publication bias with a symmetrical inverted funnel (Figure S1). The P values of Begg’s test and Egger’s test were 0.051 and 0.058 respectively. In East Asian populations, we identify a significant genetic heterogeneity with *I^2^* > 50% and *P* = 0.051. Therefore, we calculated the OR value using random effect model. The results of meta-analysis did not indicate any association between rs744373 and AD risk with *P* = 0.391, OR = 1.06, and 95% CI 0.93-1.21 (Table 3). In Caucasian populations, there were not a genetic heterogeneity among the studies with *I^2^* = 33% and *P* = 0.054. We then used the fixed effect model and calculated a significant overall OR value (*P* = 5.99 × 10^-11^, OR = 1.20, and 95% CI 1.14-1.27).

1. Results for the dominant model

For the recessive model, we did not identify any significant genetic heterogeneity among pooled Populations with *I^2^* = 49.9% and *P* = 0.002. Therefore, we calculated the OR value using random effect model. The results of meta-analysis indicated a significant association between rs744373 and AD risk in pooled populations with *P* = 1.35 × 10^-05^, OR = 1.19, and 95% CI 1.10-1.29 (Table 3). We did not identify publication bias with a symmetrical inverted funnel (Figure S2). The P values of Begg’s test and Egger’s test were 0.850 and 0.973 respectively. In East Asian populations, we identified a significant genetic heterogeneity with *I^2^* = 67% and *P* = 0.004. Therefore, we calculated the OR value using random effect model. The results of meta-analysis did not indicate any association between rs744373 and AD risk with *P* = 0.806, OR = 1.03, and 95% CI 0.81-1.31 (Table 3). In Caucasian populations, there were not a genetic heterogeneity among the studies with *I^2^* = 36% and *P* = 0.067. We then used the fixed effect model and calculated a significant overall OR value (*P* = 1.00 × 10^-05^, OR = 1.26, and 95% CI 1.14-1.39).


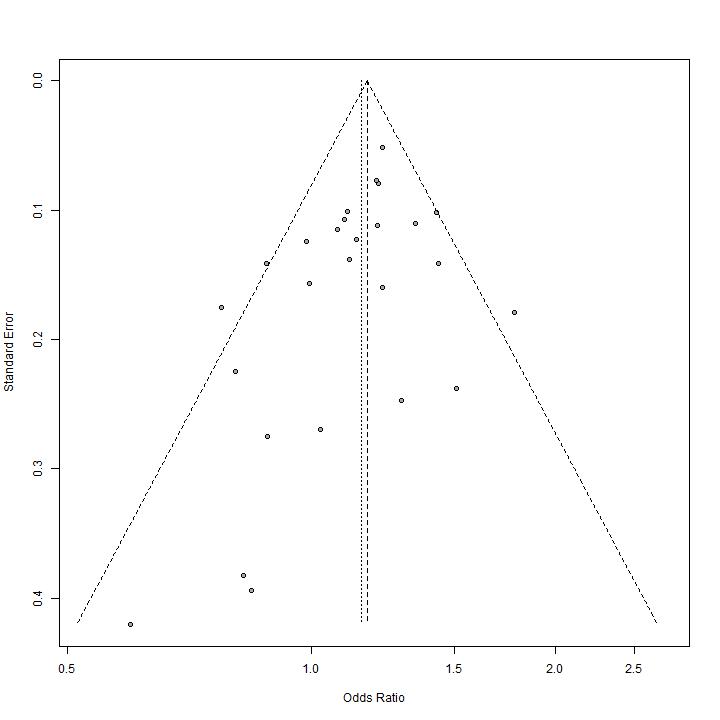


**Fig. S1.** Funnel plot of the publication bias analysis under dominant model.


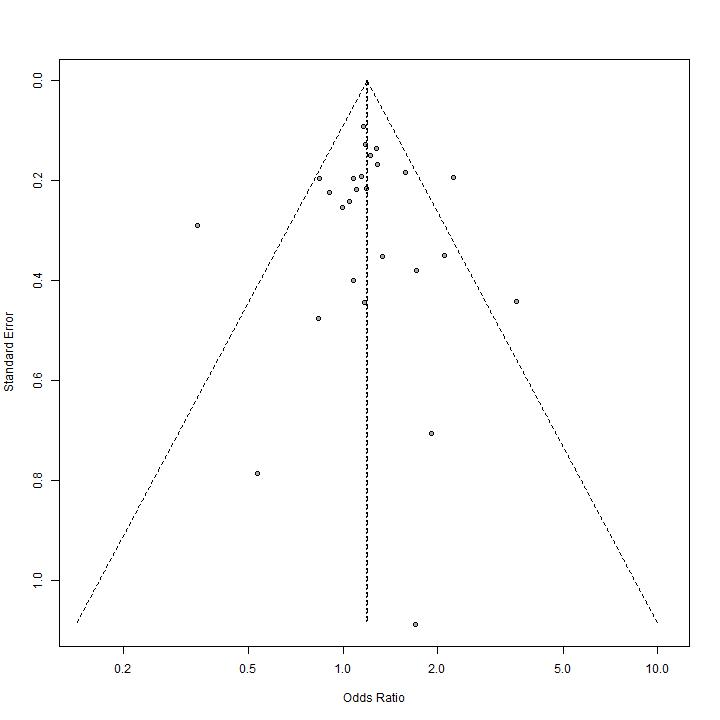


**Fig. S2.** Funnel plot of the publication bias analysis under recessive model.

**References**

1. Huang F, Shang Y, Luo Y, Wu P, Huang X, Tan X, Lu X, Zhen L, Hu X: **Lower Prevalence of Alzheimer's Disease among Tibetans: Association with Religious and Genetic Factors**. *Journal of Alzheimer's disease : JAD* 2016, **50**(3):659-667.

2. Jiao B, Liu X, Zhou L, Wang MH, Zhou Y, Xiao T, Zhang W, Sun R, Waye MM, Tang B *et al*: **Polygenic Analysis of Late-Onset Alzheimer's Disease from Mainland China**. *PloS one* 2015, **10**(12):e0144898.

3. Liao YC, Lee WJ, Hwang JP, Wang YF, Tsai CF, Wang PN, Wang SJ, Fuh JL: **ABCA7 gene and the risk of Alzheimer's disease in Han Chinese in Taiwan**. *Neurobiology of aging* 2014, **35**(10):2423 e2427-2423 e2413.

4. Tan L, Yu JT, Zhang W, Wu ZC, Zhang Q, Liu QY, Wang W, Wang HF, Ma XY, Cui WZ: **Association of GWAS-linked loci with late-onset Alzheimer's disease in a northern Han Chinese population**. *Alzheimer's & dementia : the journal of the Alzheimer's Association* 2013, **9**(5):546-553.

5. Ohara T, Ninomiya T, Hirakawa Y, Ashikawa K, Monji A, Kiyohara Y, Kanba S, Kubo M: **Association study of susceptibility genes for late-onset Alzheimer's disease in the Japanese population**. *Psychiatric genetics* 2012, **22**(6):290-293.

6. Li HL, Yang P, Liu ZJ, Sun YM, Lu SJ, Tao QQ, Guo QH, Wu ZY: **Common variants at Bin1 are associated with sporadic Alzheimer's disease in the Han Chinese population**. *Psychiatric genetics* 2015, **25**(1):21-25.

7. Wang HZ, Bi R, Hu QX, Xiang Q, Zhang C, Zhang DF, Zhang W, Ma X, Guo W, Deng W *et al*: **Validating GWAS-Identified Risk Loci for Alzheimer's Disease in Han Chinese Populations**. *Molecular neurobiology* 2016, **53**(1):379-390.

8. Carrasquillo MM, Belbin O, Hunter TA, Ma L, Bisceglio GD, Zou F, Crook JE, Pankratz VS, Sando SB, Aasly JO *et al*: **Replication of BIN1 association with Alzheimer's disease and evaluation of genetic interactions**. *Journal of Alzheimer's disease : JAD* 2011, **24**(4):751-758.

9. Lambert JC, Zelenika D, Hiltunen M, Chouraki V, Combarros O, Bullido MJ, Tognoni G, Fievet N, Boland A, Arosio B *et al*: **Evidence of the association of BIN1 and PICALM with the AD risk in contrasting European populations**. *Neurobiology of aging* 2011, **32**(4):756.e711-755.

10. Harold D, Abraham R, Hollingworth P, Sims R, Gerrish A, Hamshere ML, Pahwa JS, Moskvina V, Dowzell K, Williams A *et al*: **Genome-wide association study identifies variants at CLU and PICALM associated with Alzheimer's disease**. *Nature genetics* 2009, **41**(10):1088-1093.

11. Ramos Dos Santos L, Belcavello L, Camporez D, Iamonde Maciel de Magalhaes C, Zandonade E, Lirio Morelato R, Imbroisi Valle Errera F, Drumond Louro I, Do Carmo Pimentel Batitucci M, de Paula F: **Association study of the BIN1 and IL-6 genes on Alzheimer's disease**. *Neuroscience letters* 2016, **614**:65-69.
